# Male Breast Cancer Incidence and Mortality Risk in the Japanese Atomic Bomb Survivors – Differences in Excess Relative and Absolute Risk from Female Breast Cancer

**DOI:** 10.1289/EHP151

**Published:** 2016-06-10

**Authors:** Mark P. Little, Damien M. McElvenny

**Affiliations:** 1 Radiation Epidemiology Branch, Division of Cancer Epidemiology and Genetics, National Cancer Institute, National Institutes of Health, Department of Health and Human Services, Rockville, Maryland, USA; 2 Institute of Occupational Medicine, Edinburgh, United Kingdom

## Abstract

**Background:**

There are well-known associations of ionizing radiation with female breast cancer, and emerging evidence also for male breast cancer. In the United Kingdom, female breast cancer following occupational radiation exposure is among that set of cancers eligible for state compensation and consideration is currently being given to an extension to include male breast cancer.

**Objectives:**

We compare radiation-associated excess relative and absolute risks of male and female breast cancers.

**Methods:**

Breast cancer incidence and mortality data in the Japanese atomic-bomb survivors were analyzed using relative and absolute risk models via Poisson regression.

**Results:**

We observed significant (p ≤ 0.01) dose-related excess risk for male breast cancer incidence and mortality. For incidence and mortality data, there are elevations by factors of approximately 15 and 5, respectively, of relative risk for male compared with female breast cancer incidence, the former borderline significant (p = 0.050). In contrast, for incidence and mortality data, there are elevations by factors of approximately 20 and 10, respectively, of female absolute risk compared with male, both statistically significant (p < 0.001). There are no indications of differences between the sexes in age/time-since-exposure/age-at-exposure modifications to the relative or absolute excess risk. The probability of causation of male breast cancer following radiation exposure exceeds by at least a factor of 5 that of many other malignancies.

**Conclusions:**

There is evidence of much higher radiation-associated relative risk for male than for female breast cancer, although absolute excess risks for males are much less than for females. However, the small number of male cases and deaths suggests a degree of caution in interpretation of this finding.

**Citation:**

Little MP, McElvenny DM. 2017. Male breast cancer incidence and mortality risk in the Japanese atomic bomb survivors – differences in excess relative and absolute risk from female breast cancer. Environ Health Perspect 125:223–229; http://dx.doi.org/10.1289/EHP151

## Introduction

Female breast cancer is the most commonly occurring cancer among women in developed and developing regions of the world (WHO 2015). Male breast cancer is much rarer—the number of incident cases of male breast cancer is typically about 0.5–1% of the number of female breast cancers in many developed western populations (Landis et al. 1999; ONS 2012). There is a similar ratio of male breast cancer deaths to female breast cancer deaths (ONS 2004). Male and female breast cancer share some etiological features, although not all (Weiss et al. 2005).

Female breast cancer has been associated with exposure to moderate and high doses (> 100 mGy) of ionizing radiation in the Japanese atomic bomb survivors Life Span Study (LSS) cohort and in women who received radiotherapy (UNSCEAR 2008). A pooled analysis of eight cohorts suggested that excess relative risks of female breast cancer are (dependent on cohort) modified by age at exposure or attained age (Preston et al. 2002). There is emerging evidence to suggest that male breast cancer may also be radiogenic, in the LSS incidence data set (Ron et al. 2005) and in a population-based U.S case–control study (Thomas et al. 1994). However, possibly due to the small number of cases, Ron et al. (2005) did not report analyses of exposure response trend. There has been no similar study of male breast cancer in the latest LSS mortality follow-up (Ozasa et al. 2012).

In the United Kingdom, the Industrial Injuries Advisory Council (IIAC 2015) is currently considering amending the list of cancers arising from occupational exposure to ionizing radiation for which state compensation may be claimed, if exposure is sufficient to double the relative risk of disease. Included within these considerations is whether or not to recommend male breast cancer should be added to the list that currently includes female breast cancer: This has provided the motivation for this further analysis. Male breast cancer is currently regarded as a disease for which compensation can be paid if the probability of causation is sufficiently high by the U.S. Department of Labor, and the same relative risk model is used for both sexes (U.S. Department of Labor 2016). However, the U.S. National Cancer Institute RadRAT probability of causation calculation software does not have a model for male breast cancer (Berrington de Gonzalez et al. 2012).

In this paper, we analyze male and female breast cancer incidence and mortality in the latest versions of the LSS incidence (Preston et al. 2007) and mortality data (Ozasa et al. 2012). We assess the statistical comparability of measures of generalized excess relative risk and excess absolute risk between males and females, specifically focusing on dose–response trends and their modification by attained age, and age at exposure. Such generalized excess relative and absolute risk models have previously been shown to provide a good description of breast cancer risk in the LSS and in other radiation-exposed groups (Little and Boice 1999; Preston et al. 2002). We shall emphasize estimates of excess relative risk because of their ready applicability to estimate probability of causation (IAEA 1996).

## Methods

### Study Population and Data Sources

The LSS breast cancer incidence data used is the publicly available version of the data set analyzed by Preston et al. (2007). Details of the study population and methods have been published previously (Preston et al. 2007). Their analysis assessed cancer incidence over the years 1958–1998 in the two cities (Hiroshima and Nagasaki, Japan), and this should be roughly comparable with follow-up in the earlier publication of Ron et al. (2005); however, total numbers of cases differ slightly [see Supplemental Material A Table A2, and Table 1 of Ron et al. (2005)], which we discuss later. Likewise, the breast cancer mortality data set is the publicly available version of the data set analyzed by Ozasa et al. (2012). This analysis assessed mortality over the years 1950–2003. Summary numbers of breast cancer cases, deaths, and person years by sex are given in Table 1. Unless otherwise stated, analysis is restricted to those resident in either city (Hiroshima, Nagasaki) at the time of the bombings and to those with known breast dose.

**Table 1:** Summary information on numbers of breast cancer cases and deaths by sex in the LSS incidence (Preston et al. 2007) and mortality (Ozasa et al. 2012) data.

Type of data	Males	Females	Total
Incidence data^*a*^
Cases	7	847	854
Persons	32,411	47,769	80,180
Mean age at exposure (years) (range)^*b*^	20.58 (< 0.41, > 78.59)	24.29 (< 0.37, > 81.58)	22.91 (< 0.37, > 81.58)
Mean attained age (years) (range)^*b*^	50.19 (< 13.90, > 107.14)	54.53 (< 13.89, > 108.44)	52.91 (< 13.89, > 108.44)
Mean dose (Sv) (range)^*b*^^,^^*c*^	0.15 (0, > 5.46)	0.14 (0, > 4.78)	0.15 (0, > 5.46)
Person years (PY)	778,687	1,305,300	2,083,987
Rate (/10^5^ PY)	0.90	64.89	40.98
Mortality data
Deaths	6	324	330
Persons	35,687	50,924	86,611
Mean age at exposure (years) (range)^*b*^	20.28 (< 0.45, > 85.74)	23.77 (< 0.06, > 88.63)	22.41 (< 0.06, > 88.63)
Mean attained age (years) (range)^*b*^	47.87 (< 7.80, > 112.14)	52.15 (< 7.60, > 113.29)	50.49 (< 7.60, > 113.29)
Mean dose (Sv) (range)^*b*^^,^^*c*^	0.15 (0, > 5.45)	0.15 (0, > 5.33)	0.15 (0, > 5.45)
Person years (PY)	1,280,800	2,013,490	3,294,290
Rate (/10^5^ PY)	0.47	16.09	10.02
^***a***^Persons who were in either city, with known dose. ^***b***^Person-year weighted mean. ^***c***^DS02 breast dose, Sv, using neutron relative biological effectiveness of 10.			

### Statistical Methods

Poisson regression methods were used to investigate breast cancer risks. A linear relative risk model was fitted in which the expected number of deaths in stratum *i* (defined by certain grouped values of city, sex, attained age, and age at exposure) and dose group *d* with mean breast dose *D_id_* (in Sv), sex *s* (∈{*m*, *f*}) and mean attained age *a_id_*, age at exposure *e_id_* and time since exposure *t_id_* (all in years) is given in Model 1:

*PY_id_*λ*_i_*(1 + α*_s_D_id_*exp[β_1_[*a_id_* – 50]/10 + β_2_[*t_id_* – 30]/10+β_3_[*e_id_* – 20]/10]) [1]

where *PY_id_* is the number of person years of follow-up. λ*_i_* is the expected cancer rate in stratum *i*, and α*_s_* is the excess relative risk (ERR)/Sv, both estimated from the model fit, along with all other model parameters (β_1_, β_2_, β_3_). The values of 50, 20, 30 years subtracted from the attained age, age at exposure, and time since exposure are the approximate mean values of these variables in the two data sets; we did this to stabilize parameter estimates. Similar models of breast cancer risk have been fitted previously to these and other breast cancer data sets (Little and Boice 1999; Preston et al. 2002; UNSCEAR 2008). A slight generalization of this model was also fitted (Model 2), allowing for the adjustment parameters β_1_, β_2_, β_3_ to vary by sex *s*∈{*m*, *f*}:

*PY_id_*λ*_i_*(1 + α*_s_D_id_*exp[β_1_ *_s_*[*a_id_* – 50]/10 + β_2_ *_s_*[*t_id_* – 30]/10 + β_3_ *_s_*[*e_id_* – 20]/10]) [2]

The neutron component of breast cancer dose incorporates a weighting factor (relative biological effectiveness) of 10, to account for the known higher effectiveness of this type of radiation compared with that of high energy gamma rays (International Commission on Radiological Protection 2007).

We also evaluated the excess absolute risk (EAR), modeling that required us to construct a parametric function of the baseline (zero dose) risks. We assumed that the expected number of cases or deaths in stratum *i* with certain values of explanatory variables (*Z_idj_*)*^N^_j_*_=_ *_1_* (e.g., city, sex, age, time since exposure) is given in Model 3:

*PY_id_*(*f*((*Z_idj_*)*^N^_j_*_= 1_,(γ*_j_*)*^N^_j_*_= 1_) + α*_s_D_id_*exp[β_1_[*a_id_* – 50]/10 *+* β_2_[*t_id_* – 30]/10 + β_3_[*e_id_* – 20]/10]) [3]

or analogous to Model 2, we also consider Model 4:

*PY_id_*(*f*((*Z_idj_*)*^N^_j_*_= 1_,(γ*_j_*)*^N^_j_*_= 1_) + α*_s_D_id_*exp[β_1_ *_s_*[*a_id_* – 50]/10 *+* β_2_ *_s_*[*t_id_* – 30]/10 + β_3_ *_s_*[*e_id_* – 20]/10]) [4]

Here the baseline cancer rate is given by Model 5:


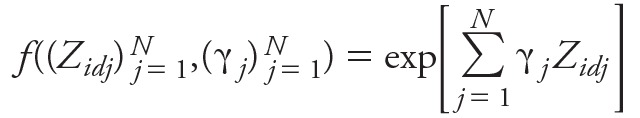
_[5]_

a function of the explanatory variables and some parameters, (γ*_j_*)*^N^_j_*_=_ *_1_*, the latter determined by the model fit; the contrast with the semi-parametric rates, λ*_i_*, in Models 1 and 2 should be noted. In order to adequately fit breast cancer incidence and mortality, taking account of all factors other than radiation in the two data sets, we considered models for *f*() constructed from a candidate set of variables that included city, sex, all terms ln[*age*/50]*^k^*, ln[years since exposure/30]*^k^*, and [age at exposure – 20]*^k^* with integral *k* taking values between 1 and 6, and all second order interactions of these (e.g., terms of the form ln[*age*/50]^3^ × ln[years since exposure/30]^6^). In order to avoid over-parameterized models, the Akaike Information Criterion (AIC) (Akaike 1973, 1981) was employed to select the optimal subset of descriptive variables from this set. AIC penalizes against overfitting by adding 2 × [number of fitted parameters] to the model deviance. A mixed forward-backward stepwise algorithm was used to select the set of variables minimizing AIC, using R (version 3.2.2; R Project for Statistical Computing). The indicated optimal models were augmented to make them polynomially complete, so that if the optimal model included a variable *A^M^B^N^* for some indices 1 ≤ *M*, *N* ≤ 6, then all terms *A^m^B^n^* for indices 0 ≤ *m* ≤ *M*, 0 ≤ *n* ≤ *N* were also included in the model. The final set of variables in the optimal models for breast cancer incidence and mortality are listed in Supplemental Material B Table B1. Models with parametrically modeled baseline rates of the sort given by Model 4 but using a relative risk formulation were also fitted:

*PY_id_*(*f*((*Z_idj_*)*^N^_j_*_= 1_,(γ*_j_*)*^N^_j_*_= 1_) (1 + α*_s_D_id_*exp[β_1_ *_s_*[*a_id_* – 50]/10 + β_2_ *_s_*[*t_id_* – 30]/10 + β_3_ *_s_*[*e_id_* – 20]/10]) [6]

However, these models are in some ways less flexible than the Models 1 and 2 with semi-parametrically modeled baseline rates, and in particular cannot be readily fitted to the male breast cancer data by itself, because there are too few cases. Results are generally similar to those using the semi-parametric relative risk models, so we shall not refer to them in this article.

In Models 1 to 4, we are mainly interested in the excess risk coefficients, 〈*_s_*, and in the temporally modifying parameters (by attained age, time since exposure, age at exposure), β_1_, β_2_, β_3_. Notice that *t_id_* = *a_id_* – *e_id_* so we only fit submodels of Models 1 to 4 with at most two of the three parameters β_1_, β_2_, β_3_ (or β_1s_, β_2s_, β_3s_) allowed to be nonzero. All model parameters are estimated via Poisson maximum likelihood (McCullagh and Nelder 1989), using Epicure (version 2.0.1.0; Risk Sciences International). All hypothesis tests are based on the likelihood-ratio test, and unless otherwise stated confidence intervals (CIs) were based on the profile likelihood (McCullagh and Nelder 1989).

If cancer rate following assumed radiation dose *D* is given by *f*((*Z_j_*)*^N^_j_*_= 1_,(γ*_j_*)*^N^_j_*_= 1_) + *g*(*D*)*h*((*Z_j_*)*^N^_j_*_= 1_,(γ*_j_*)*^N^_j_*_= 1_) for some functions *f*(), *g*(), *h*(), then the probability of causation (PC) associated with radiation is given in Model 7:

*g*(*D*)*h*((*Z_j_*)*^N^_j_*_= 1_,(γ*_j_*)*^N^_j_*_= 1_)/ {*f*((*Z_j_*)*^N^_j_*_= 1_,(γ*_j_*)*^N^_j_*_= 1_) + *g*(*D*)*h*((*Z_j_*)*^N^_j_*_= 1_,(γ*_j_*)*^N^_j_*_= 1_)} _[7]_

Further details on the rationale are given elsewhere (IAEA 1996). In particular, when the model is of relative risk form as in Model 1, this simplifies to Model 8:

α*_s_D_id_*exp[β_1_[*a_id_* – 50]/10 + β_2_[*t_id_* – 30]/10 + β_3_[*e_id_* – 20]/10]/ {1 + α*_s_D_id_*exp[β_1_[*a_id_* – 50]/10 + β_2_[*t_id_* – 30]/10 + β_3_[*e_id_* – 20]/10]} [8]

We estimated PC for male breast cancer using the model fitted here and compared it with PC estimated for various other sites, using relative risk models fitted by the UNSCEAR (2008) and by the Biological Effects of Ionizing Radiation VII (BEIR VII) committee (NRC Committee to Assess Health Risks from Exposure to Low Levels of Ionizing Radiation 2006) to current LSS data, in Supplemental Material A Table A1.

We evaluated PC for attained age of 65 years, which is approximately the median age of occurrence for male breast cancer in the LSS, assuming exposure to 50 mSv at 35 and 55 years old. Although 50 mSv is about twice the mean lifetime cumulative dose, 24.9 mSv, in the United Kingdom’s National Registry for Radiation Workers, there are 20,373 workers (11.7% of the cohort) with cumulative doses above this level (Muirhead et al. 2009).

## Results

There are a total of 7 incident cases of male breast cancer, and 6 male breast cancer deaths (Table 1). There are 847 female breast cancer cases, and 324 female breast cancer deaths, which are greater than the corresponding figures for men by factors of ~ 100 to ~ 50, respectively. Crude breast cancer incidence rates are higher by a factor of ~ 70 (64.89/0.90) among women than men, and breast cancer mortality rates are higher by a factor of ~ 34 (16.09/0.47) (Table 1). In Table 2, we show that most cases and deaths are > 60 years old (4/7 cases, 5/6 deaths). Most cases and deaths also occur among the younger age at exposure groups, with exposure age < 40 years old (5/7 cases, 5/6 deaths). There are highly statistically significant trends with dose for male breast cancer incidence (*p* = 0.003) (Table 3). The breast cancer incidence ERR for males adjusted for the effect of attained age and age at exposure is 27.68 Sv^–1^ (95% CI: 1.81, 90.16) higher by a factor of ~ 15 than the analogous trend risk of 1.86 Sv^–1^ (95% CI: 1.36, 2.46) for females (Table 3). This difference is borderline statistically significant (*p* = 0.050) (Table 3). These results are much the same without adjustment for the modifying effects (on the ERR) of attained age and age at exposure. In Table 4, we demonstrate that the optimal adjustment to EAR is for time since exposure. Table 4 shows that the EAR for males (normalized to 30 years after exposure) is 0.38/10^4^ person-year Sv (95% CI: 0.07, 0.89); whereas for females, the EAR is higher by a factor of ~ 20, 7.25/10^4^ person-year Sv (95% CI: 5.53, 9.13), a difference that is highly statistically significant (*p* < 0.001). The comparison of EARs between the sexes is much the same without adjustment for time since exposure (Table 4).

**Table 2:** Male breast cancer cases and deaths, and person years of follow-up, by age at exposure and attained age, using data of Preston et al. (2007) and Ozasa et al. (2012).

Age at exposure (years)	Breast cancer incident cases/deaths Attained age (years)						Person years Attained age					
	0–39	40–49	50–59	60–69	70–79	≥ 80	0–39	40–49	50–59	60–69	70–79	≥ 80
Those in either city with known dose (incident cases)
0–19	0	1	2	0	0		231816	115956	95032.7	37203.2	1303.64
20–39	0	0	0	1	1	0	4759.08	29054.8	51543.6	43488.9	27530.9	7270.12
40–49			0	1	0	1			13154.6	39802	25719.2	11321
50–59				0	0	0				9918.88	19097.3	7365.45
60–69					0	0					3177.86	3703.17
≥ 70						0						468.543
Those in either city, possibly with unknown dose (incident cases)
0–19	0	1	2	0	0		246570	124531	102503	41222.1	1476.71
20–39	0	0	0	1	2	0	5593.58	33507.8	58876.6	49815.2	31504.5	8223.99
40–49			0	1	0	2			14604.5	43735.8	28250.9	12297.5
50–59				0	0	0				10859.6	20725.7	7944.78
60–69					0	0					3337.86	3823.55
≥ 70						0						496.141
Those in either city with known dose (deaths)
0–19	0	1	0	1	1		418150	156749	147267	82930.8	15448.1
20–39	0	0	0	0	1	1	25876.4	55010.5	56421.7	47740.6	34072.7	13716.7
40–49		0	0	0	0	1		5996.07	46511.2	44057.8	28841.2	13658.9
50–59			0	0	0	0			5796.36	31886.5	21362.1	8146.53
60–69				0	0	0				2878.1	11670.6	4166.76
≥ 70					0	0					754.569	1685.79

**Table 3:** Breast cancer incidence risk by sex, using stratified relative risk Model 1 with strata defined by city, sex, age at exposure, attained age, using data of Preston et al. (2007).

Model number	Model/parameter fitted	ERR/Sv (95% CI)	Temporal modifiers (95% CI)	Deviance	*p*-Value^*a*^
1	Male breast cancer (α_*m*_)	19.41 (1.53, 761.30)	—	53.95	0.003^*b*^
2	Female breast cancer (α_*f*_)	1.50 (1.12, 1.95)	—	2770.49	< 0.001^*b*^
3	Male and female breast cancer (α)	1.54 (1.15, 1.98)	—	2828.32	< 0.001^*b*^
		—	—	—	0.049^*c*^
4	Adjusted for attained age (α)	1.90 (1.41, 2.50)	—	2820.14	0.004
	Attained age adjustment (per 10 years of age) (exp[β_1_])	—	0.70 (0.52, 0.90)	—	—
5	Adjusted for time since exposure (α)	1.62 (1.20, 2.13)	—	2827.37	0.331^*d*^
	Time since exposure adjustment (per 10 years of time since exposure) (exp[β_2_])	—	0.89 (0.70, 1.13)	—	—
6	Adjusted for age at exposure (α)	1.59 (1.19, 2.05)	—	2825.28	0.081^*d*^
	Age at exposure adjustment (per 10 years of age at exposure) (exp[β_3_])	—	0.82 (0.64, 1.02)	—	—
7	Adjusted for attained age and age at exposure (α)	1.89 (1.39, 2.49)	—	2820.07	0.016^*d*^
	Attained age adjustment (per 10 years of age) (exp[β_1_])	—	0.71 (0.52, 0.95)	—	0.023^*e*^
	Age at exposure adjustment (per 10 years of age at exposure) (exp[β_3_])	—	0.97 (0.73, 1.26)	—	0.802^*f*^
8	Adjusted for sex, attained age and age at exposure: males (α_*m*_)	27.68 (1.81, 90.16^*g*^)	—	2816.24	0.050
	Adjusted for sex, attained age and age at exposure: females (α_*f*_)	1.86 (1.36, 2.46)	—	—	—
	Attained age adjustment (per 10 years of age) (exp[β_1_])	—	0.70 (0.51, 0.94)	—	—
	Age at exposure adjustment (per 10 years of age at exposure) (exp[β_3_])	—	0.99 (0.75, 1.30)	—	—
Note: ––, data not available. ^***a***^Unless otherwise indicated the *p*-value represents the improvement in fit over the model in the row immediately above it; all model numbers refer to the leftmost column. ^***b***^*p*-Value of improvement in fit over the null model, with no terms in dose. ^***c***^*p*-Value of improvement in fit of model allowing for different dose coefficients (α_*m*_, α_*f*_) by sex, over Model 3, in other words what is obtained by comparing the combined fit of Models 1 and 2 with that of Model 3. ^***d***^*p*-Value of improvement in fit over Model 3. ^***e***^*p*-Value of improvement in fit over Model 6. ^***f***^*p*-Value of improvement in fit over Model 4. ^***g***^Wald-based CI.					

**Table 4:** Breast cancer incidence risk by sex, using absolute risk Model 3, using data of Preston et al. (2007).

Model number	Model/parameter fitted	EAR/10^4^ person year Sv (95% CI)	Temporal modifiers (95% CI)	Deviance	*p*-Value^*a*^
1	Null model	—	—	3168.32	—
2	Male and female breast cancer (α)	2.36 (1.49, 3.36)	—	3119.66^*b*^	< 0.001
3	Male breast cancer (α_*m*_)	0.35 (0.02, 0.88)	—	3054.49	< 0.001
	Female breast cancer (α_*f*_)	7.07 (5.38, 8.92)	—	—	—
4	Adjusted for attained age (α)	2.28 (1.38, 3.30)	—	3118.49	0.279^*c*^
	Attained age adjustment (per 10 years of age) (exp[β_1_])	—	1.12 (0.91, 1.40)	—	—
5	Adjusted for time since exposure (α)	2.38 (1.45, 3.43)	—	3114.00	0.017^*c*^
	Time since exposure adjustment (per 10 years of time since exposure) (exp[β_2_])	—	1.46 (1.07, 2.02)	—	—
6	Adjusted for age at exposure (α)	2.40 (1.51, 3.43)	—	3119.49	0.676^*c*^
	Age at exposure adjustment (per 10 years of age at exposure) (exp[β_3_])	—	0.95 (0.75, 1.19)	—	—
7	Adjusted for attained age and time since exposure (α)	2.37 (1.41, 3.44)	—	3114.00	0.059^*c*^
	Attained age adjustment (per 10 years of age) (exp[β_1_])	—	1.01 (0.79, 1.29)	—	0.933^*d*^
	Time since exposure adjustment (per 10 years of time since exposure) (exp[β_2_])	—	1.45 (1.03, 2.08)	—	0.034^*e*^
8	Adjusted for sex, time since exposure: males (α_*m*_)	0.38 (0.07, 0.89)	—	3042.84^*b*^	< 0.001^*d*^
	Adjusted for sex, time since exposure: females (α_*f*_)	7.25 (5.53, 9.13)	—	—	—
	Time since exposure adjustment (per 10 years of time since exposure) (exp[β_2_])	—	1.41 (1.16, 1.71)	—	—
Note: ––, data not available. ^***a***^Unless otherwise indicated the *p*-value represents the improvement in fit over the model in the row immediately above it; all model numbers refer to the leftmost column. ^***b***^Indications of lack of convergence. ^***c***^*p*-Value of improvement in fit over Model 2. ^***d***^*p*-Value of improvement in fit over Model 5. ^***e***^*p*-Value of improvement in fit over Model 4.					

There are highly statistically significant trends with dose for male breast cancer mortality (*p* = 0.010) (Table 5). The breast cancer mortality ERR for males adjusted for the effect of attained age and age at exposure is 9.48 Sv^–1^ (95% CI: 0.38, 154.90), higher by a factor of ~ 5 than the analogous trend risk of 1.86 Sv^–1^ (95% CI: 1.36, 2.46) for females (Table 5). This difference is not statistically significant (*p* > 0.2) (Table 5). As for the incidence data, these results are much the same without adjustment for the modifying effects (on the ERR) of attained age and age at exposure. We demonstrate that the optimal temporal adjustment to EAR is for time since exposure. Table 6 shows that the EAR for males (normalized to 30 years after exposure) is 0.16/10^4^ person-year Sv (95% CI: 0.02, 0.39); whereas for females, the EAR is higher by a factor of ~ 10, 1.53/10^4^ person-year Sv (95% CI: 0.86, 2.31), a difference that is highly statistically significant (*p* < 0.001). The comparison of EARs between the sexes is much the same without adjustment for time since exposure (Table 6).

**Table 5:** Breast cancer mortality risk by sex, using stratified relative risk Model 1, with strata defined by city, sex, age at exposure, attained age, using data of Ozasa et al. (2012).

Model number	Model/parameter fitted	ERR/Sv (95% CI)	Temporal modifiers (95% CI)	Deviance	*p*-Value^*a*^
1	Male breast cancer (α_*m*_)	8.88 (0.60, 92.34)	—	48.34	0.010^*b*^
2	Female breast cancer (α_*f*_)	1.56 (0.96, 2.34)	—	1804.10	< 0.001^*b*^
3	Male and female breast cancer (α)	1.64 (1.02, 2.42)	—	1854.49	< 0.001^*b*^
		—	—	—	0.152^*c*^
4	Adjusted for attained age (α)	2.10 (1.21, 3.35)	—	1852.21	0.131
	Attained age adjustment (per 10 years of age) (exp[β_1_])	—	0.79 (0.54, 1.07)	—	—
5	Adjusted for time since exposure (α)	1.37 (0.72, 2.19)	—	1851.91	0.108^*d*^
	Time since exposure adjustment (per 10 years of time since exposure) (exp[β_2_])	—	1.26 (0.95, 1.76)	—	—
6	Adjusted for age at exposure (α)	1.78 (1.03, 2.72)	—	1842.94	< 0.001^*d*^
	Age at exposure adjustment (per 10 years of age at exposure) (exp[β_3_])	—	0.54 (0.33, 0.79)	—	—
7	Adjusted for attained age and age at exposure (α)	1.85 (0.93, 3.10)	—	1842.90	0.003^*d*^
	Attained age adjustment (per 10 years of age) (exp[β_1_])	—	0.96 (0.62, 1.43)	—	0.840^*e*^
	Age at exposure adjustment (per 10 years of age at exposure) (exp[β_3_])	—	0.55 (0.33, 0.82)	—	0.002^*f*^
8	Adjusted for sex, attained age and age at exposure: males (α_*m*_)	9.48 (0.38, 154.90)	—	1841.51	0.239
	Adjusted for sex, attained age and age at exposure: females (α_*f*_)	1.86 (0.96, 3.10)	—	—	—
	Attained age adjustment (per 10 years of age) (exp[β_1_])	—	0.92 (0.58, 1.38)	—	—
	Age at exposure adjustment (per 10 years of age at exposure) (exp[β_3_])	—	0.57 (0.34, 0.85)	—	—
Note: ––, data not available. ^***a***^Unless otherwise indicated the *p*-value represents the improvement in fit over the model in the row immediately above it; all model numbers refer to the leftmost column. ^***b***^*p*-Value of improvement in fit over the null model, with no terms in dose. ^***c***^*p*-Value of improvement in fit of the model allowing for different dose coefficients (α_*m*_, α_*f*_) by sex, over Model 3, in other words what is obtained by comparing the combined fit of Models 1 and 2 with that of Model 3. ^***d***^*p*-Value of improvement in fit over Model 3. ^***e***^*p*-Value of improvement in fit over Model 6. ^***f***^*p*-Value of improvement in fit over Model 4.					

**Table 6:** Breast cancer mortality risk by sex, using absolute risk Model 3, using data of Ozasa et al. (2012).

Model number	Model/parameter fitted	EAR/10^4^ person year/Sv (95% CI)	Temporal modifiers (95% CI)	Deviance	*p*-Value^*a*^
1	Null model	—	—	2221.65	—
2	Male and female breast cancer (α)	0.58 (0.27, 0.97)	—	2203.91^*b*^	< 0.001
3	Male breast cancer (α_*m*_)	0.13 (–0.04^*c*^, 0.43)	—	2185.32	< 0.001
	Female breast cancer (α_*f*_)	1.63 (0.98, 2.40)	—	—	—
4	Adjusted for attained age (α)	0.57 (0.24, 0.98)	—	2198.63	0.022^*d*^
	Attained age adjustment (per 10 years of age) (exp[β_1_])	—	1.37 (1.05, 1.88)	—	—
5	Adjusted for time since exposure (α)	0.53 (0.17, 0.96)	—	2185.18^*b*^	< 0.001^*d*^
	Time since exposure adjustment (per 10 years of time since exposure) (exp[β_2_])	—	2.11 (1.50, 3.48)	—	—
6	Adjusted for age at exposure (α)	0.59 (0.24, 0.98)	—	2202.94	0.324^*d*^
	Age at exposure adjustment (per 10 years of age at exposure) (exp[β_3_])	—	0.83 (0.48, 1.19)	—	—
7	Adjusted for attained age and time since exposure (α)	0.53 (0.18, 0.97)	—	2185.07^*b*^	< 0.001^*d*^
	Attained age adjustment (per 10 years of age) (exp[β_1_])	—	0.94 (0.63, 1.35)	—	0.745^*e*^
	Time since exposure adjustment (per 10 years of time since exposure) (exp[β_2_])	—	2.20 (1.43, 3.87)	—	< 0.001^*f*^
8	Adjusted for sex, time since exposure: males (α_*m*_)	0.16 (0.02, 0.39)	—	2159.70	< 0.001^*e*^
	Adjusted for sex, time since exposure: females (α_*f*_)	1.53 (0.86, 2.31)	—	—	—
	Time since exposure adjustment (per 10 years of time since exposure) (exp[β_2_])	—	1.83 (1.45, 2.40)	—	—
Note: ––, data not available. ^***a***^Unless otherwise indicated the *p*-value represents the improvement in fit over the model in the row immediately above it; all model numbers refer to the leftmost column. ^***b***^Indications of lack of convergence. ^***c***^Wald-based CI. ^***d***^*p*-Value of improvement in fit over Model 2. ^***e***^*p*-Value of improvement in fit over Model 5. ^***f***^*p*-Value of improvement in fit over Model 4.					

Models were also fitted that allowed for separate adjustments by sex for age, time since exposure or age at exposure using relative and absolute risk models. In general, there was no evidence of such heterogeneity by sex, except for a borderline significant (*p* = 0.094) difference between the time since exposure trends in the mortality relative risk data, with males exhibiting a strong decrease in risk over time compared with a modest increase over time for females [adjustment per decade of time since exposure of 0.11 and 1.26 respectively (data not shown)] (Table 7).

**Table 7:** Evidence of variation by sex in the modifying adjustment to the ERR or EAR by attained age, time since exposure, age at exposure in the breast cancer incidence and mortality data of Preston et al. (2007) and Ozasa et al. (2012), respectively. Breast cancer risk modeled using Models 2 and 4.

Type of fitted model	*p*-Value^*a*^	
	Incidence data	Mortality data
Relative risk Model 2 with univariate adjustment for either: *a*) attained age; *b*) time since exposure; or *c*) age at exposure.
Model adjusted for sex, attained age × sex compared with model adjusted for sex, attained age only.	> 0.2^*b*^	0.206
Model adjusted for sex, time since exposure × sex compared with model adjusted for sex, time since exposure only.	0.816	0.094
Model adjusted for sex, age at exposure × sex compared with model adjusted for sex, age at exposure only.	0.499	0.715
Relative risk Model 2 with adjustment for attained age, age at exposure.
Model adjusted for sex, age at exposure, attained age × sex compared with model adjusted for sex, age at exposure, attained age.	> 0.2^*b*^	> 0.2^*b*^
Model adjusted for sex, age at exposure × sex, attained age compared with model adjusted for sex, age at exposure, attained age.	0.462	0.685
Model adjusted for sex, age at exposure × sex, attained age × sex compared with model adjusted for sex, age at exposure, attained age.	> 0.2^*b*^	> 0.1^*b*^
Absolute risk Model 4 with adjustment for time since exposure.
Model adjusted for sex, time since exposure × sex compared with model adjusted for sex, time since exposure.	> 0.2^*b*^	> 0.2^*b*^
^***a***^The *p*-value represents the improvement in fit over the model with specified temporal adjustments and with adjustment for sex in the linear dose coefficients (α_*m*_,α_*f*_) obtained by adding interactions by gender to the temporal modification terms. ^***b***^Indications of lack of convergence.		

## Discussion

The analyses of this paper suggest that male breast cancer has an ERR that exceeds that for female breast cancer. This elevation of male relative risk compared to female is particularly strong (and borderline statistically significant, *p* = 0.05) for breast cancer incidence, where it is higher by a factor of ~ 15, but the elevation is also quite pronounced for breast cancer mortality, higher by a factor of ~ 5 (but not statistically significant, *p* > 0.2). However, the male breast cancer absolute excess risks are lower by a factor of ~ 10 to ~ 20 than those for females (and highly statistically significant, *p* < 0.001), reflecting the much lower baseline cancer rates for males than for females.

Indeed, the findings of a high ratio of male:female relative excess risks of breast cancer (*ERR_male_/ERR_female_*), and low ratio of male:female absolute excess risks (*EAR_male_/EAR_female_*), is largely accounted for by the ratio of male:female baseline breast cancer rates (*CR_male_/CR_female_*). Comparison of Models 1 and 3 would lead one to expect that it is approximately the case that:

*ERR_male_/ERR_female_ ≈* (*EAR_male_/CR_male_*)*/*(*EAR_female_/CR_female_*) *≈* (*EAR_male_/EAR_female_*)(*CR_female_/CR_male_*) [9]

The adequacy of this approximation may be judged by the fact that the left-hand side of Model 9 is 27.68/1.86 = 14.88 for incidence (Table 3) and 9.48/1.86 = 5.10 for mortality (Table 5), while the right-hand side can be estimated by (0.38/7.25)[(320/575,694)/(1/342,504)] = 9.98 for incidence (Table 4; see also Supplemental Material A Table A2) and (0.16/1.53)[(119/893,939)/(2/571,320)] = 3.98 for mortality (Table 6; see also Supplemental Material A Table A3).

Because of the much lower EARs of male compared with female breast cancer, our findings imply minimal impact on assessments of individual or population breast cancer risk following all but therapeutic levels of radiation exposure, compared with those using models proposed by national (NRC Committee to Assess Health Risks from Exposure to Low Levels of Ionizing Radiation 2006) and international (UNSCEAR 2008) radiation safety committees. Nevertheless, they imply potentially substantial probabilities of causation following modest (e.g., occupational) radiation exposure (IAEA 1996). The analysis of Supplemental Material A Table A1 suggests that male breast cancers following moderate (occupational) exposure, of 50 mSv, whether incurred at exposure age 35 or 55 years, would be associated with a PC of about 44%, at least five times more than the PC associated with most other highly radiogenic cancer sites, in particular leukemia, and cancers of the stomach, colon, female breast, and brain and central nervous system. However, the calculations for male breast cancer are subject to substantial uncertainties, as may be deduced from the width of the confidence intervals in Table 3. In principle, EAR models could also be used to evaluate PC. However, the particular models we developed are not useful for evaluating this quantity. Because an adequate model of breast cancer in the baseline (unexposed) population would necessarily have to incorporate terms for city (Hiroshima, Nagasaki), it makes them difficult to apply in any context other than to this particular cohort.

There are no strong indications of differences between the sexes in the temporal modifications (by attained age, time since exposure, age at exposure). This is perhaps a function of lack of statistical power due to the very small numbers of cases and deaths in males. There are borderline significant indications that time since exposure modifications in relative risk may differ (*p* = 0.09) between the sexes, with the male ERR concentrated in the earlier years of follow-up compared with the female. This does not contradict the pattern shown in Supplemental Material A Table A3, which shows that, as one would expect, all (radiogenic and other) male breast cancer cases are overwhelmingly concentrated in the later years of follow-up, a simple consequence of the ageing of the cohort.

A detailed comparison of the number of cases and person years of follow-up in the incidence data set we used and that in the paper of Ron et al. (2005) highlights some slight differences [see Supplemental Material A Table A2, and Table 1 of Ron et al. (2005)]. In particular, Ron et al. (2005) appear to have an extra case in the lowest breast dose group (0.005–0.5 Sv), we suspect because Ron et al. were using an early (and not completely validated) version of the incidence data that was later published by Preston et al. (2007). The comparison of numbers of breast cancer deaths (see Supplemental Material A Table A3) and incident cases (see Supplemental Material A Table A2) by dose group suggests that the breast cancer deaths and incident cases are somewhat different—indeed at least two of the breast cancer deaths cannot have been in the incidence data set, while at least three of the incident cases could not have been in the mortality data. Mortality in the LSS is ascertained for those remaining resident in Japan, while incidence is restricted to those people resident in the two cities. There are also temporal differences in follow-up (for mortality 1950–2003, for incidence 1958–1998). This could account for the deaths that do not appear to be incident cases. Given that all the male breast cancer cases occur relatively late (after 1971) (see Supplemental Material A Table A4), when effective treatments for breast cancer (male and female) became available, it is quite likely that there will be people who develop breast cancer who do not die from it, thereby accounting for the cancer cases not in the mortality data. Nevertheless, one cannot entirely exclude the possibility that there are errors in the data, and as we discussed at the top of the paragraph in relation to the discrepancies in person year and case counts in the 0.005–0.5 Sv dose group, there is some evidence of this in the data of Ron et al. (2005), which we do not use.

There are very few other studies of male breast cancer in relation to exposure to ionizing radiation. A large U.S. case–control study of male breast cancer, using cases diagnosed from 10 Surveillance, Epidemiology and End Results (SEER) registries, evaluated ionizing radiation as a risk factor, and observed a trend for an increasing risk of breast cancer with an increasing number of self-reported radiographic examinations, which was statistically significant for exams performed between 1933 and 1963, although not for any later period (1964–1987) (Thomas et al. 1994). After radiation therapy, a marginally elevated risk was observed for men first treated in the period 1940–1954, and the risk was somewhat higher when the location of the treatment field resulted in exposure to the breast (Thomas et al. 1994). Evaluation of age and time effects was limited: age at radiation exposure was not statistically significantly related to breast cancer risk, and risk was increased only 20–35 years after radiation exposure. The study has major weaknesses, acknowledged by the authors (Thomas et al. 1994), in particular the low response rate, particularly among the controls (selected by random digit dialing), and the interview-based assessment of past exposures, which may be subject to recall bias. The lack of any estimates of radiation dose, whether due to diagnostic or therapeutic procedures, and the small number of exposed individuals also limit the causal interpretation of these findings.

Women experience menarche and menopause, which are not experienced by men, and the timing of these events appear to influence both the baseline risk of breast cancer (Collaborative Group on Hormonal Factors in Breast Cancer 2012) and its sensitivity to radiation induction (Land et al. 1994). Other risk factors for male breast cancer overlap somewhat with those for women, and include obesity and lack of physical activity (Brinton et al. 2008); however, the lack of risk associated with alcohol consumption (Brinton et al. 2008) is in striking contrast to the consistent risk seen in relation to increased alcohol consumption for female breast cancer (Baan et al. 2007; Cao et al. 2015). Germline mutations in the *BRCA2* gene are a risk factor for both male and female breast cancer, but mutations in the *BRCA1* gene appears to be a risk much more for female breast cancer than for male breast cancer (Ford et al. 1998; Greene 1997; Rizzolo et al. 2013). Mutations in the *PTEN* gene have also been linked to both male and female breast cancer (Fackenthal et al. 2001; Marsh et al. 1998), as additionally have mutations in *CHEK2* (Nevanlinna and Bartek 2006). Male breast cancer has been genetically linked with the *AR* gene (Lobaccaro et al. 1993; Wooster et al. 1992). The differences in male breast cancer etiology that we highlight may have some bearing on the fact that male breast cancer radiation-associated relative risk appears to be substantially higher than that of women, and the weak indications (*p* = 0.094) that time since exposure modifications in relative risk may differ between the sexes. However, the small number of cases and deaths in the data sets that we have analyzed argues for a degree of caution in interpretation of this finding.

Nevertheless, our findings build on those of Ron et al. (2005) in suggesting that male breast cancer incidence and mortality is radiogenic, with a degree of ERR that is at least as large as that for female breast cancer. As such, there is a case for the IIAC (2015) and other similar bodies to consider recommending the inclusion of male breast cancer in the list of cancers arising from occupational exposure to ionizing radiation for which compensation may be claimed, as is indeed already the case in the United States (U.S. Department of Labor 2016).

## Supplemental Material

(131 KB) PDF
