# Supplementary material for: Male Breast Cancer Incidence and Mortality Risk in the Japanese Atomic Bomb Survivors – Differences in Excess Relative and Absolute Risk from Female Breast Cancer
Source: Environ Health Perspect. 2016 Jun 10;125(2):223–9. doi: 10.1289/EHP151 (PMC5289903; doi:10.1289/EHP151)
Supplement: (131 KB) PDF [file EHP151.s001.acco.pdf]

Environ Health Perspect
DOI: 10.1289/EHP151

Note to readers with disabilities: *EHP* strives to ensure that all journal content is accessible to all readers. However, some figures and Supplemental Material published in *EHP* articles may not conform to 508 standards due to the complexity of the information being presented. If you need assistance accessing journal content, please contact ehp508@niehs.nih.gov. Our staff will work with you to assess and meet your accessibility needs within 3 working days.

Supplemental Material

Male Breast Cancer Incidence and Mortality Risk in the Japanese Atomic Bomb Survivors – Differences in Excess Relative and Absolute Risk from Female Breast Cancer

M.P. Little and D.M. McElvenny

Table of Contents

Supplemental Material A. Supplemental Analysis Tables

Table A1. Percent probability of causation of incident case of various types of cancer at age 65, associated with 50 mSv received at ages 35 and 55, using relative risk models developed by UNSCEAR (United Nations Scientific Committee on the Effects of Atomic Radiation (UNSCEAR) 2008), BEIR VII (Committee to Assess Health Risks from Exposure to Low Levels of Ionizing Radiation 2006), and those of the present paper.

Table A2. Breast cancer cases and person years (PY) of follow-up by dose group, and whether not-in-city (NIC) survivors are included or not.

Table A3. Breast cancer deaths and person years (PY) of follow-up by dose group

Table A4. Male breast cancer cases and deaths and person years (PY) of follow-up by interval of follow-up.

Supplemental Material B. Variables used for background models of breast cancer incidence and mortality in fits of generalized excess absolute risk model

Table B1. Optimal variables used in baseline models, selected to minimize Akaike Information Criterion (AIC), and augmented to make polynomially complete.

Supplemental Material A. Supplemental Analysis Tables

Table A1. Percent probability of causation of incident case of various types of cancer at age 65, associated with 50 mSv received at ages 35 and 55, using relative risk models developed by UNSCEAR (United Nations Scientific Committee on the Effects of Atomic Radiation (UNSCEAR) 2008), BEIR VII (Committee to Assess Health Risks from Exposure to Low Levels of Ionizing Radiation 2006), and those of the present paper.

	**Age at exposure 35**		**Age at exposure 55**	
Type of incident cancer case/risk models	**Male**	**Female**	**Male**	**Female**
**UNSCEAR 2006 models**				
Stomach	1.60	1.60	1.60	1.60
Colon	2.84	2.84	2.84	2.84
Lung	1.57	6.54	1.57	6.54
Brain and central nervous system	1.07	1.07	0.69	0.69
Leukemia	4.46	4.46	4.46	4.46
Breast		4.65	0.00	4.65
**BEIR VII models**				
Stomach	0.92	2.08	0.92	2.08
Colon	2.71	1.87	2.71	1.87
Lung	1.40	5.83	1.40	5.83
Brain and central nervous system[a]	1.05	1.73	1.05	1.73
Leukemia	4.96	5.38	8.01	8.67
Breast		2.09	0.00	2.09
**Breast (present paper, table 3, model 8)**	44.51	2.99	44.00	2.95

[a]using model for all other solid cancers

Table A2. Breast cancer cases and person years (PY) of follow-up by dose group, and whether not-in-city (NIC) survivors are included or not.

		Excluding NIC		Including NIC	
Breast dose category (Sv)	Mean breast dose (Sv)	Cases	PY	Cases	PY
		Males			
0 - <0.005	0.0006	1	342,504	3	604,098
0.005 - <0.5	0.0988	4	375,300	4	375,300
0.5 - <1.0	0.7038	1	26,731	1	26,731
≥1.0	1.8620	1	34,152	1	34,152
Dose unknown	NA	2	71,213	2	71,213
		Females			
0 - <0.005	0.0007	320	575,694	546	994,844
0.005 - <0.5	0.1023	379	631,045	379	631,045
0.5 - <1.0	0.7047	51	50,885	51	50,885
≥1.0	1.8006	97	47,680	97	47,680
Dose unknown	NA	101	103,418	101	103,418

Table A3. Breast cancer deaths and person years (PY) of follow-up by dose group

Breast dose category (Sv)	Mean breast dose (Sv)	Deaths	PY
	Males		
0 - <0.005	0.0011	2	571,320
0.005 - <0.5	0.0981	1	610,627
0.5 - <1.0	0.7158	2	43,653
≥1.0	1.8766	1	55,197
	Females		
0 - <0.005	0.0011	119	893,939
0.005 - <0.5	0.1016	142	964,406
0.5 - <1.0	0.7093	23	79,559
≥1.0	1.8108	40	75,581

Table A4. Male breast cancer cases and deaths and person years (PY) of follow-up by interval of follow-up.

	Incidence		Mortality	
Interval of follow-up	Cases	PY	Deaths	PY
<1961/1/1	0	84,545	0	342,982
1961/1/1 - 1970/12/31	0	246,762	0	288,140
1971/1/1 - 1980/12/31	2	195,952	0	243,573
1981/1/1 - 1990/12/31	4	154,809	2	201,845
1991/1/1 +	1	96,618	4	204,257

Supplemental Material B. Variables used for background models of breast cancer incidence and mortality in fits of generalized excess absolute risk model

Table B1. Optimal variables used in baseline models, selected to minimize Akaike Information Criterion (AIC), and augmented to make polynomially complete.

**Breast cancer incidence**
city
sex
ln[age/50], ln[age/50]^{2}, ln[age/50]^{3}, ln[age/50]^{4}, ln[age/50]^{5}, ln[age/50]^{6}
ln[years since exposure/30], ln[years since exposure/30]^{2}
[age at exposure - 20], [age at exposure - 20]^{2}
city*ln[age/50], city*ln[age/50]^{2}, city*ln[age/50]^{3}, city*ln[age/50]^{4}
city*ln[years since exposure/30] city*ln[years since exposure/30]^{2}
ln[years since exposure/30]*[age at exposure - 20], ln[years since exposure/30]*[age at exposure - 20]^{2}
ln[age/50]*ln[years since exposure/30], ln[age/50]^{2}*ln[years since exposure/30], ln[age/50]^{3}*ln[years since exposure/30], ln[age/50]^{4}*ln[years since exposure/30]
ln[age/50]*ln[years since exposure/30]^{2}, ln[age/50]^{2}*ln[years since exposure/30]^{2}, ln[age/50]^{3}*ln[years since exposure/30]^{2}, ln[age/50]^{4}*ln[years since exposure/30]^{2} [a]
**Breast cancer mortality**
city
sex
ln[age/50], ln[age/50]^{2}, ln[age/50]^{3}
ln[time since exposure/30], ln[time since exposure/30]^{2}, ln[time since exposure/30]^{3}
ln[age/50]*ln[time since exposure/30], ln[age/50]*ln[time since exposure/30]^{2}
ln[age/50]^{2}*ln[time since exposure/30], ln[age/50]^{2}*ln[time since exposure/30]^{2}
sex*ln[age/50], sex*ln[age/50]^{2} [b]
sex*ln[time since exposure/30]
city*ln[time since exposure/30]

[a] ln[age/50]^{4}*ln[years since exposure/30]^{2} term caused problems of convergence, and was dropped from the model.

[b] sex*ln[age/50]^{2} term caused problems of convergence, and was dropped from the model.

References

Committee to Assess Health Risks from Exposure to Low Levels of Ionizing Radiation NRC. 2006. Health risks from exposure to low levels of ionizing radiation: Beir vii - phase 2. Washington, DC, USA:National Academy Press.

United Nations Scientific Committee on the Effects of Atomic Radiation (UNSCEAR). 2008. Unscear 2006 report. Annex a. Epidemiological studies of radiation and cancer. New York:United Nations, 13-322.
